# Alectinib, an anaplastic lymphoma kinase (ALK) inhibitor, as a bridge to allogeneic stem cell transplantation in a patient with ALK‐positive anaplastic large‐cell lymphoma refractory to chemotherapy and brentuximab vedotin

**DOI:** 10.1002/ccr3.2543

**Published:** 2019-11-15

**Authors:** Ritsuko Nakai, Suguru Fukuhara, Akiko Miyagi Maeshima, Sung‐Won Kim, Yuta Ito, Shunsuke Hatta, Tomotaka Suzuki, Sayako Yuda, Shinichi Makita, Wataru Munakata, Tatsuya Suzuki, Dai Maruyama, Koji Izutsu

**Affiliations:** ^1^ Department of Hematology National Cancer Center Hospital Tokyo Japan; ^2^ Department of Pathology National Cancer Center Hospital Tokyo Japan; ^3^ Department of Hematopoietic Stem Cell Transplantation National Cancer Center Hospital Tokyo Japan

**Keywords:** alectinib, ALK‐positive ALCL, hematopoietic stem cell transplantation

## Abstract

This is the first case report of alectinib as a bridge to allo‐SCT in a patient with ALK‐positive ALCL refractory to both conventional chemotherapies and BV. This report offers a ray of hope for a condition with poor prognosis.

## Graphical abstract

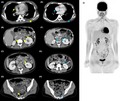

## 1 INTRODUCTION

A patient with ALK‐positive anaplastic large‐cell lymphoma was refractory to both conventional chemotherapies and brentuximab vedotin, but responded to alectinib, leading to allogeneic stem cell transplantation with complete response. Alectinib may become a promising treatment option for such patients.

Anaplastic lymphoma kinase (ALK)‐positive anaplastic large‐cell lymphoma (ALCL) is known to have a better prognosis than other peripheral T‐cell lymphomas (PTCLs),^1^ including ALK‐negative ALCL, but relapsed or refractory patients with ALCL had poor outcomes before the brentuximab vedotin (BV) era, regardless of ALK status.^2^ There is some evidence that high‐dose chemotherapy and autologous stem cell transplantation (HDC/ASCT) or allogeneic stem cell transplantation (allo‐SCT) may offer long‐term benefits for patients with relapsed or refractory ALCL.^3^ BV, which is an antibody–drug conjugate consisting of an anti‐CD30 monoclonal antibody and monomethyl auristatin E, showed a high rate of durable remissions in ALCL patients regardless of ALK status and has also been evaluated as a bridging agent to transplantation.^4^ Meanwhile, a small retrospective study reported that patients who experienced progressive disease while receiving BV had poor outcomes.^5^ Here, we report a patient with ALK‐positive ALCL who was refractory to both conventional chemotherapies and BV but who responded to alectinib, leading to allo‐SCT with metabolic complete response.

## 2 CASE PRESENTATION

The patient was a 22‐year‐old female who was admitted to our hospital via a primary care hospital. She had a persistent high fever despite receiving a systemic corticosteroid, as well as worsening low back pain, paralytic ileus, and paresis of the lower limbs. Her Eastern Cooperative Oncology Group performance status (PS) was 4. She reported analgesia below the level of the 10th thoracic vertebra and demonstrated weakness of the quadriceps and triceps muscles. Laboratory tests showed a white blood cell count of 22.4 × 10^9^/L with no atypical lymphocytes, a hemoglobin concentration of 9.7 g/dL, a platelet count of 8.5 × 10^9^/L, a lactate dehydrogenase concentration of 1396 IU/L, and a soluble interleukin‐2 receptor concentration of 115 259 IU/L. Contrast computed tomography (CT) revealed cervical and abdominal lymphadenopathy in addition to an anterior chest wall mass, bilateral pleural effusion, hepatosplenomegaly, and multiple bone lesions. Biopsy of the anterior chest wall mass and bone marrow examination showed infiltration by large, CD30‐positive lymphoid cells, consistent with ALCL with nuclear and cytoplasmic expression of ALK. Given these clinical findings, the patient was diagnosed with ALK‐positive ALCL, Ann Arbor clinical stage IV, and high risk according to the International Prognostic Index (IPI).

**Figure 1:**
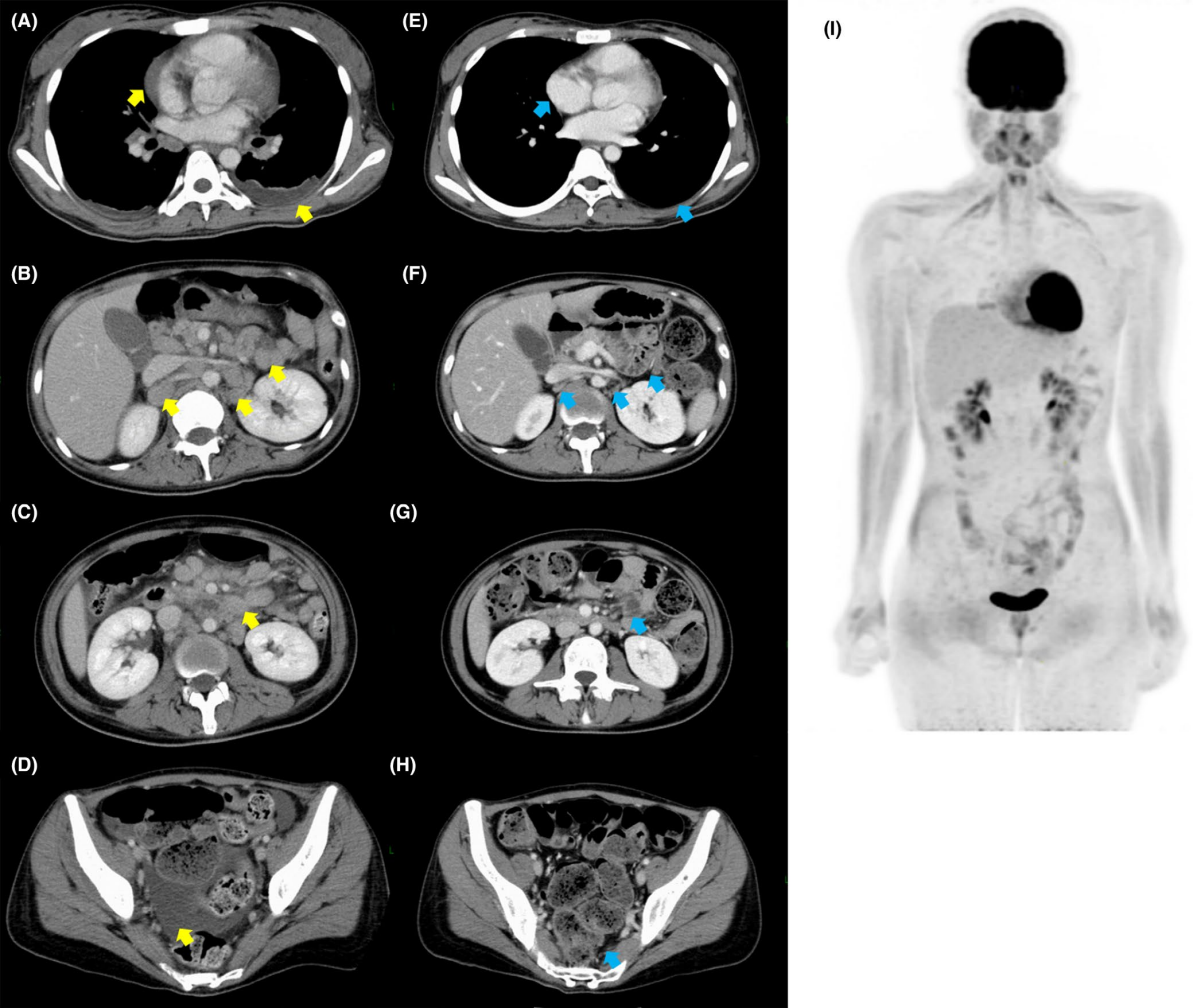
A‐D, CT images before treatment with alectinib show bilateral pleural effusion, pericardial effusion, ascites, and multiple lymph node enlargement (yellow arrows). E‐H, CT images after treatment with alectinib (day 12) show disappearance of bilateral pleural effusion, pericardial effusion, ascites, and multiple lymph node enlargement (blue arrows). I, FDG‐PET/MRI images after treatment with alectinib (day 24) show no abnormal uptake

Standard chemotherapy with CHOP (cyclophosphamide, doxorubicin, vincristine, and prednisone) was started as the first‐line treatment. At the same time, the patient received radiotherapy to the thoracic spine (30 gray [Gy] in 10 fractions) to alleviate the spinal cord compression causing lower extremity paresis. Her pyrexia and low back pain temporarily improved, but after a second course of CHOP, new lesions appeared in the bilateral axillary lymph nodes and right hip joint. We planned salvage chemotherapy followed by ASCT for primary refractory ALK‐positive ALCL. We initiated the ESHAP regimen (etoposide, methylprednisolone, cytarabine, and cisplatin) as salvage therapy, though we had to discontinue this treatment due to anaphylaxis to cisplatin on day 1. BV monotherapy (1.8 mg/kg every 3 weeks) was initiated as the third‐line treatment, but disease progression was noted after the second course. BV with CHP (cyclophosphamide, doxorubicin, and prednisolone) as the fourth regimen was also ineffective, and new lesions emerged in the patient's right ileum and femur at the end of second course, with severe pain requiring opioids and palliative radiotherapy. A CT scan showed worsened bilateral pleural effusion, pericardial effusion, ascites, and enlargement of multiple lymph nodes (Figure 1A‐D).

At this point, we initiated the off‐label use of alectinib, an ALK inhibitor, at 300 mg twice daily. Written informed consent from the patient and approval of the institutional committee for off‐label use was obtained. After starting alectinib treatment, the patient demonstrated rapid daily improvement. On day 2, she was afebrile, and her pain was markedly decreased. She was able to discontinue opioid intake on day 12; a CT scan that day showed no ascites or lymphadenopathy (Figure 1E‐H). Finally, she was discharged from the hospital on day 13. The adverse effects (AEs) of alectinib were dysgeusia, skin eruptions on the upper limbs, pretibial edema, and a slight elevation of aspartate aminotransferase and alanine aminotransferase (grade 1 according to the Common Terminology Criteria for Adverse Events [CTCAE] Version 4.0). There were no increases in bilirubin or alkaline phosphatase. All AEs were manageable and resolved spontaneously within a few weeks. On day 24, complete metabolic response was demonstrated by fluoro‐deoxy‐glucose (FDG)–positron emission tomography (PET)/magnetic resonance imaging (MRI) (Figure 1I).

As the patient had no human leukocyte antigen (HLA)–identical sibling donor, she underwent allogeneic bone marrow transplantation from an unrelated HLA‐1‐locus–mismatched female donor following a reduced‐intensity conditioning regimen consisting of fludarabine 30 mg/m^2^ daily for 6 consecutive days (total 180 mg/m^2^), busulfan 3.2 mg/kg daily for 2 consecutive days (total 6.4 mg/kg), and total body irradiation (2 Gy in one fraction). She continued taking alectinib until the day before starting the conditioning regimen. She achieved successful engraftment and complete chimerism. No acute transplant‐related complications were experienced except acute graft‐vs‐host disease of the skin (stage 1). At 23 months after transplantation, she remains in complete response (CR) and has not required further treatment for refractory ALK‐positive ALCL.

## 3 DISCUSSION

Generally, ALK‐positive ALCL patients have a better clinical outcome.^1^ However, the survival of relapsed or refractory patients is very poor.^2^ For these patients, the current treatment strategy is salvage chemotherapy followed by either auto‐SCT or allo‐SCT in fit patients.^6^ Although conventional salvage chemotherapy regimens have been used, such as ESHAP, GDP (gemcitabine, dexamethasone, and cisplatin), and ICE (ifosfamide, carboplatin, and etoposide), the efficacy of these multidrug combination chemotherapies has not been satisfactory.^7^, ^8^, ^9^ By contrast, BV has significantly improved the long‐term outcomes of patients with relapsed or refractory ALCL, regardless of ALK status. In a pivotal phase II study, the overall response rate (ORR) and CR rate were 86% and 57%, respectively.^10^ The median progression‐free survival (PFS) was 13.3 months, which was significantly longer than that achieved with prior therapies. BV has also been evaluated as a bridging agent to transplantation.^4^ However, a retrospective study showed that the median OS after BV failure was only 2.9 months.^5^ This fact indicates a high unmet need for new treatment strategies for patients with BV‐refractory ALCL.

Crizotinib is a small‐molecule ALK inhibitor that has demonstrated high antitumor efficacy in ALK‐positive tumors, including non–small‐cell lung cancer (NSCLC).^11^ Crizotinib has also shown significant activity in pediatric patients with ALK‐positive ALCL.^11^ In a phase I study that was part of a larger trial of crizotinib in ALK‐positive malignancies, the ORR and CR rate were 88% (8 of 9) and 78% (7 of 9), respectively.^11^ Trials of crizotinib in adults are ongoing, both as a first‐line therapy and for relapsed or refractory disease (NCT02487316, NCT01524926). Alectinib is a potent oral selective tyrosine kinase inhibitor of ALK that demonstrates antitumor activity against cancers with ALK gene alterations. In enzymatic assays, alectinib was approximately 5 times more potent than crizotinib against ALK and inhibited most of the clinically observed acquired ALK resistance mutations to crizotinib.^12^ Alectinib showed superior efficacy and lower toxicity than crizotinib in the primary treatment of ALK‐positive NSCLC.^13^, ^14^ Therefore, we chose alectinib 300 mg twice daily as it is the approved dose for ALK‐positive NSCLC in Japan.^14^ Alectinib showed high antitumor efficacy in this case, and it appears to be an optimal bridging therapy to SCT for BV‐refractory, ALK‐positive ALCL patients. However, it is not obvious whether treatment with alectinib alone can result in long‐term survival without SCT in this population. In a previous study, 3 of 7 patients remained in CR for over 1 year at the time of analysis (16, 23, and 28 months).^11^ Conversely, it was reported that long‐term use of alectinib caused acquired resistance responsible for relapse in NSCLC patients.^15^ In the present case, we chose to proceed to allo‐SCT while the patient was in CR. Recently, a case was reported in which alectinib showed high efficacy in a chemotherapy intolerant, ALK‐positive ALCL patient.^16^ A clinical trial of alectinib for ALK‐positive ALCL is ongoing in Japan (UMIN000016991). We expect that further clinical research will elucidate the efficacy and safety of ALK inhibitors in ALCL patients.

In conclusion, this is the first case report of the use of alectinib as bridging therapy to allo‐SCT in a patient with ALK‐positive ALCL refractory to both conventional chemotherapy and BV. Although the roles of ALK inhibitors in ALK‐positive ALCL remain a matter of debate, alectinib may become a viable therapeutic option for patients with relapsed or refractory ALCL following treatment with BV and chemotherapy.

## CONFLICT OF INTEREST

Dr Nakai, Dr Fukuhara, Dr Maeshima, Dr Kim, Dr Ito, Dr Hatta, Dr Tomotaka Suzuki, Dr Yuda, Dr Makita, and Dr Munakata have nothing to disclose. Dr Izutsu reports the following funding outside the submitted work: grants from Eisai, grants and personal fees from Kyowa Hakko Kirin, grants and personal fees from MSD, grants and personal fees from Takeda, grants and personal fees from Janssen, personal fees from Bristol Myers Squib, personal fees from Dainihon Sumitomo, grants and personal fees from Mundipharma, personal fees from Nihon Mediphysics, grants and personal fees from Chugai, personal fees from Astra Zeneca, grants and personal fees from Abbvie, grants and personal fees from Bayer, grants and personal fees from Ono, grants from Gilead, grants from Zenyaku, grants and personal fees from Celgene, grants from Solasia, grants from Symbio, grants from Astellas, grants from Astellas Amgen, grants from Daiichi Sankyo. Dr Maruyama reports grants and personal fees from Chugai, grants and personal fees from Takeda during the conduct of the study, and the following funding outside the submitted work: grants from Sanofi, grants and personal fees from Janssen, grants and personal fees from Eisai, grants and personal fees from Celegene, grants and personal fees from Kyowa Hakko Kirin, grants and personal fees from Ono, grants and personal fees from Mundipharma, grants and personal fees from MSD, grants and personal fees from Zenyaku, personal fees from Sumitomo, personal fees from Asahi Kasei, grants and personal fees from Bristol‐Myers Squibb, grants and personal fees from Daiichi Sankyo, grants and personal fees from AstraZeneca, personal fees from Linical, personal fees from Pharma International, personal fees from Fujimoto, grants from Abbvie, grants from Astellas, grants from Amgen Astellas Biopharma, grants from Otsuka, grants from Novartis, grants from Pfizer, grants from Solasia, grants from Bayer, grants from Symbio, grants from CMIC, grants from Quintiles, grants from IQvia. Dr Tatsuya Suzuki reports personal fees from Chugai Pharmaceutical, outside the submitted work.

## AUTHOR CONTRIBUTIONS

RN SF: designed this project. KI: supervised this project. RN, SF, SK, YI, SH, ToS, and YS: managed the patient. AM: performed immunohistological evaluation. RN and SF: wrote the preliminary draft of the manuscript. SM, WT, TaS, DM, and KI: critically revised the manuscript. All authors wrote and reviewed subsequent of manuscript and approved the final version.
